# Contribution of Tennis Involvement to Successful Aging: The Case of Masters Tennis Players

**DOI:** 10.3390/sports14010017

**Published:** 2026-01-04

**Authors:** Elif Bozyiğit, Şeniz Karagöz, István Karsai, Gusztáv József Tornóczky

**Affiliations:** 1 Department of Sport Management, Faculty of Sport Science, Pamukkale University, 20160 Denizli, Türkiye; ebozyigit@pau.edu.tr; 2 Department of Recreation, Faculty of Sport Science, Afyon Kocatepe University, 03200 Afyonkarahisar, Türkiye; skaragoz@aku.edu.tr; 3 Physical Education and Exercise Centre, Medical School, University of Pecs, 7624 Pécs, Hungary; tornoczky.gusztav@aok.pte.hu; 4 Institute of Health Promotion and Sport Sciences, Faculty of Education and Psychology, ELTE Eötvös Loránd University, 1117 Budapest, Hungary

**Keywords:** successful aging, healthy lifestyle, tackling problems, leisure time involvement, tennis

## Abstract

One of the most prominent topics in contemporary research is how individuals can adopt behaviors and attitudes that support successful aging (SA) throughout their life course. Participation in sport is widely regarded as an important behavioral strategy that contributes to physical, psychological, and social resources relevant to SA. This study examined the association between Tennis Involvement (TI) and orientations toward successful aging in a sample of 224 masters tennis players with a mean age of approximately 51 years. Data was collected using the Tennis Involvement Scale and the Successful Aging Scale, which was applied to assess strategies and predispositions related to successful aging rather than aging outcomes. A structural model was tested using descriptive statistics, reliability analysis, correlation analysis, Confirmatory Factor Analysis (CFA), and Structural Equation Modeling (SEM). The hypotheses assumed that all factors of tennis involvement would be positively correlated with SA, “tackling problems-TP”, and “healthy lifestyle-HL”. However, the SEM analysis results provided partial support for hypotheses H1a and H1c. Only the “social bonding” factor showed a significant and positive correlation. Remarkably, however, the “centrality” factor showed a negative trend, contrary to expectations, and statistically significant correlations were found. No significant correlations were identified between age, TI, and SA. However, there were positive and significant correlations between players’ weekly tennis playing time (both in days and hours) and SA, TP, and HL. In conclusion, the results indicate that, among middle-aged masters tennis players, tennis involvement is associated with both positive and negative aspects of successful aging, and longer tennis playing duration is associated with more favorable successful aging strategies.

## 1. Introduction

In recent years, global and national health authorities have intensified their efforts to promote sports participation for health benefits [1,2,3]. Despite these initiatives, however, the rates of unhealthy or unsuccessful aging continue to climb [4,5]. This trend is significantly influenced by technological advancements and the increasing prevalence of sedentary lifestyles.

Understanding the role of sports participation in promoting healthier aging has become increasingly significant. Aging is a lifelong process that commences at birth and is characterized by physical, emotional, and mental regression. This process encompasses the period during which the human lifespan approaches completion, vital functions progressively decline, and the quality of life diminishes over time. Successful aging is defined as the process of developing and maintaining functional abilities that enable older individuals to perform tasks of personal importance, despite unforeseen medical conditions and accidents, while preserving independence, vitality, and quality of life [1,6].

According to the literature, several factors of human life can contribute to successful aging, such as physiological, psychological, and social domains [7], as well as additional lifestyle factors [8]. According to the framework by Rowe and Kahn [9,10], successful aging is defined by three key criteria: (1) avoiding disease and disability; (2) maintaining high cognitive and physical function; (3) sustaining meaningful engagement with life.

To age successfully, individuals should develop regular physical activity and exercise habits early in life, as these form the foundation for successful aging in both the short and long term [11,12]. It has been reported that regular exercise enhances insulin sensitivity and offers protective benefits for lipid metabolism [13]. Notably, endurance exercises help maintain vascular function in older age and reduce mortality [14,15,16]. Recent research indicates that high-intensity interval training (HIIT) is another effective method for preventing coronary heart disease, boosting cardiorespiratory fitness, and improving the cardiovascular risk profile in older adults [17].

Tennis is a widely enjoyed sport that attracts players of all ages worldwide. Its widespread appeal is bolstered by the Masters Tour system, which organizes tournaments from MT100 to MT1000 across diverse age groups at the international level [18]. As a multifaceted sport, tennis offers players physical, mental, and psychological benefits. The game’s structure, marked by short, high-intensity rallies and brief recovery periods, boosts both aerobic and anaerobic endurance [19]. Additionally, playing tennis demands a mental framework that fosters strategic thinking, concentration, and stress-management skills [20]. Long-term and regular engagement in tennis has been shown in the literature to promote healthy living and even extend life expectancy [21,22]. An epidemiological study involving 8577 individuals tracked over 25 years found that tennis players lived an average of 9.7 years longer than sedentary individuals [23]. These benefits are attributed to the blend of aerobic, strength, balance, and flexibility components of the sport, which help mitigate the risk of falls, sarcopenia, osteoporosis, and cognitive decline [21,22]. These effects are believed to stem from the multifaceted nature of tennis, which simultaneously supports the physical, cognitive, emotional, and social domains associated with successful aging [10].

Masters tennis players are known to participate in training and matches of this multifaceted nature during their leisure time [18]. Leisure time involvement is characterized by factors such as “centrality,” “identity expression,” and “attraction” [24], as well as “social bonding” and “identity affirmation” [25]. Kyle et al. [25,26] explained these factors as follows: Attraction refers to the perceived interest in an activity and the pleasure derived from participation [25,26]. Centrality examines the centrality of an activity in an individual’s life. Social bonding refers to the social ties that provide attachment to activities. Identity affirmation provides opportunities for self-affirmation through engaging in leisure activities. Identity expression provides opportunities for individuals to express themselves through leisure activities [24,25,26].

Considering this conceptual framework, tennis involvement (attraction, centrality, social bonding, identity affirmation, and identity expression) is thought to be directly related to the components of Reker’s [27] successful aging model. The attraction (hedonic value) and importance of tennis motivate individuals to maintain a healthy lifestyle by engaging in regular physical activity [28]. The social relationships inherently fostered by tennis create a strong social support network, providing a critical psychological resource for tackling the challenges of aging [17,29]. Deep identification with tennis and its use as a means of expression (identity expression) provides individuals with a meaningful identity and life purpose well into later life; this is directly linked to personal growth and life satisfaction, which are the cornerstones of successful aging [19,20].

**Figure 1:**
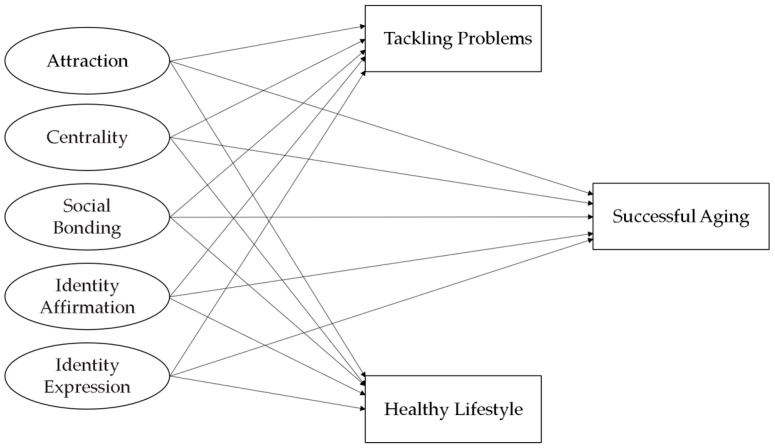
Relationships between the TI subscales and SA, TP, and HL according to the model.

In recent years, it has become evident that many individuals are focusing on various activities and lifestyle changes to enhance their quality of life and achieve a longer, healthier existence. This study aims to illuminate academic research on masters tennis players aged 40 and above, contributing to sports literature by examining health, psychological, and social relationships collectively. Consequently, this study explores the connection between tennis involvement and successful aging, with a particular focus on how regular tennis participation and competitive tournament engagement contribute to key aspects of successful aging. Based on this premise, we developed a model to investigate the relationship between tennis involvement and successful aging, as outlined in the theoretical framework presented above (Figure 1). In this context, the following hypotheses were formulated to test the model.

**H1a.**  *There are significant and positive relationships between attraction, centrality, social bonding, identity affirmation, and identity expression and successful aging.*

**H1b.**  *There are significant and positive relationships between attraction, centrality, social bonding, identity affirmation, and identity expression and tackling problems.*

**H1c.**  *There are significant and positive relationships between attraction, centrality, social bonding, identity affirmation, and identity expression and a healthy lifestyle.*

**H2.**  *There are significant correlations between the participants’ age, weekly training days and hours, and tennis involvement/subscales and successful aging/subscales.*

## 2. Materials and Methods

### 2.1. Participants and Procedure

In Türkiye, the Turkish Tennis Federation organizes official Masters Tennis competitions. Athletes must possess a valid license to participate in these tournaments. However, holding a license does not necessarily indicate that the individual is an active tennis player. Each player creates their own tournament schedule based on their personal plans. Tournaments are hosted by clubs across various provinces that meet the federation’s standards. Athletes compete in events such as the T200, T400, T500, Turkish Individual Masters Championship, and Team Championship. Since tennis is an individual sport, reaching many masters tennis players can be quite challenging. In this study, data was collected from athletes participating in masters tennis tournaments across different provinces in 2024.

The study group consisted of masters players who participated in tournaments during their leisure time, in alignment with the research purpose. Participants were interviewed individually, face-to-face, and the study’s purpose was explained within a tournament setting. Volunteer tennis players were informed about data security measures and asked to read and consent to the “informed consent form.” Instructions for completing the data collection tools were then provided, and the dataset (survey forms and pens) was distributed. The forms were anonymous, and participants could stop completing the survey at any time. Participants did not receive financial compensation for participating in the survey. Completing the scale forms took an average of 5–10 min. The researchers addressed the participants’ questions throughout the data collection period. Forms with larger font sizes were prepared for athletes experiencing vision problems at older ages and were used if necessary. Due to the difficulty in reaching the sample, incomplete or incorrect forms were identified immediately, and participants were asked to correct them, but no pressure was imposed.

Efforts were made to ensure representativeness across age groups (e.g., 40+, 45+, 50+) and gender. However, due to the significantly lower number of participants in older age groups and among female masters players, as was the case nationwide, these criteria were not adequately met. Individuals with disabilities were not included in the study. The study group comprised licensed athletes who regularly played tennis and participated in masters tournaments. The vast majority of participants are highly educated (91% university and postgraduate graduates), and the sample is more male than female (male: 68%, female: 32%). These characteristics suggest that the sample represents a non-random and hard-to-reach subgroup (individuals participating in masters tennis tournaments with high socioeconomic and cultural resources) (Table 1). Therefore, when interpreting the results, it is essential to consider that the relationships between successful aging and tennis involvement may be influenced by factors such as education and socioeconomic status, and that the results cannot be directly generalized to a broader and more heterogeneous elderly population. This study focuses on a specific, high-functioning group to examine the proactive processes of successful aging.

The project was approved by the Ethics Sub-Committee of Afyon Kocatepe University (date: 20 December 2023; meeting number: 18; decision no: 2023/388).

### 2.2. Instruments

The “Personal Information Form,” crafted by the researchers, outlined the personal and tennis-related characteristics of the participants. It includes sociodemographic factors such as gender, age, total tennis days, hours played per week, and the city where tennis is played.

#### 2.2.1. The Modified Involvement Scale (MIS)

The MIS, developed by Kyle et al. [25], was translated into Turkish as the “Leisure Involvement Scale” (LIS) by Gurbuz et al. [30], who also conducted validity and reliability studies. This scale assesses the level of involvement in leisure activities. For this study, the adaptable items from the scale were tailored for tennis and utilized as the “Tennis Involvement Scale.” For example, “A1 ______ is one of the most enjoyable things I do” was changed to “A1 Tennis is one of the most enjoyable things I do”. The scale is a 5-point Likert-type scale consisting of 15 items and five subscales. The subscales consist of the factors “attraction”, “centrality”, “social bonding”, “identity affirmation”, and “identity expression”. Each factor consisted of three items.

#### 2.2.2. The Successful Aging Scale (SAS)

The SAS was developed by Reker [27]. The original scale consisted of three subfactors and 14 items related to “healthy lifestyle,” “adaptive coping,” and “engagement with life” related to successful aging. In the study by Hazer and Özsungur [31], the scale, adapted to Turkish culture, underwent structural modifications, with non-functional items removed based on validity and reliability analyses. This scale is a 7-point Likert-type, comprising 10 items divided into two subscales: “tackling problems” and “healthy lifestyle.” The “tackling problems” subscale includes seven items, while the “healthy lifestyle” subscale contains three items.

### 2.3. Statistical Analysis

The data collected from the participants was scrutinized, with any missing or incorrect entries being removed and processed for analysis. Out of the 245 survey responses, 26 were excluded from the analysis because they either did not align with the study group characteristics or were filled out incompletely or incorrectly. Descriptive statistics were conducted to highlight the fundamental characteristics of masters tennis players (Table 1). The normal distribution of the data was assessed using skewness-kurtosis coefficients, scatter diagrams, and Mahalanobis distances, and the mean and standard deviations of the scores were calculated.

Assuming that the scales would be applied to masters tennis players for the first time, a Confirmatory Factor Analysis (CFA) was conducted to confirm that tennis involvement and successful aging are valid and reliable instruments for Turkish masters tennis players, and the goodness-of-fit values were determined. Additionally, Cronbach’s Alpha and Composite Reliability (CR) values were calculated for construct reliability, while factor loading values and Average Variance Explained (AVE) values were determined for convergent validity using CFA. For the new DFA model of scales that differed in structure from the original scale, the existing data was randomly divided in SPSS using split-half cross-validation (80–20%). A k-fold cross-validation analysis was conducted for both the exploration phase (80%) and the validation phase (20%).

The causal relationships (H1a, H1b, H1c) between observed and latent variables were tested using Structural Equation Modeling (SEM). Pearson’s Correlation analysis was performed to determine the relationships between the variables (age, TTD, and TTH) and scale scores (H_2_). During the analysis, we calculated the effect size (Cohen’s d, r^2^) indicator for the given cases. In the statistical analyses, *p* < 0.05 was considered statistically significant. SPSS 31 was used for descriptive statistics and correlation analyses, and AMOS 22 was used for CFA and SEM analyses to test the hypotheses.

## 3. Results

### 3.1. Descriptive Statistics

The information and tennis-related characteristics of masters tennis players within the scope of the research are given in Table 1.

The mean age of the study group, comprising masters tennis players over 40, was 51.23 (female max = 65, male max = 72). The participants consisted of 68% males and 32% females, with a significant number of tennis players holding university degrees. They hailed from 18 different provinces across Türkiye, including cities such as Adana, Afyonkarahisar, Ankara, Antalya, Aydın, Balıkesir, Bartın, Burdur, Bursa, Denizli, Eskişehir, İstanbul, İzmir, Kocaeli, Konya, Mersin, Muğla, and Samsun.

An analysis of the total number of tennis days and hours played showed that masters tennis players typically engage in the sport three days a week (Table 2). Interestingly, as players age, both the frequency of play and the total hours spent on the court each week tend to increase, especially among those aged 60–64. This trend may be attributed to individuals in this age bracket generally retiring from their professional lives, allowing them more time to devote to sports.

Table 3 presents the descriptive statistics and reliability coefficients for the Tennis Involvement and Successful Aging Scales.

Analyses revealed that the reliability values of the scales/subscales were moderate to high. Masters tennis players were found to agree with the statements on both measurement instruments at above-mean scores.

### 3.2. CFA Results of Tennis Involvement and Successful Aging

**Figure 2:**
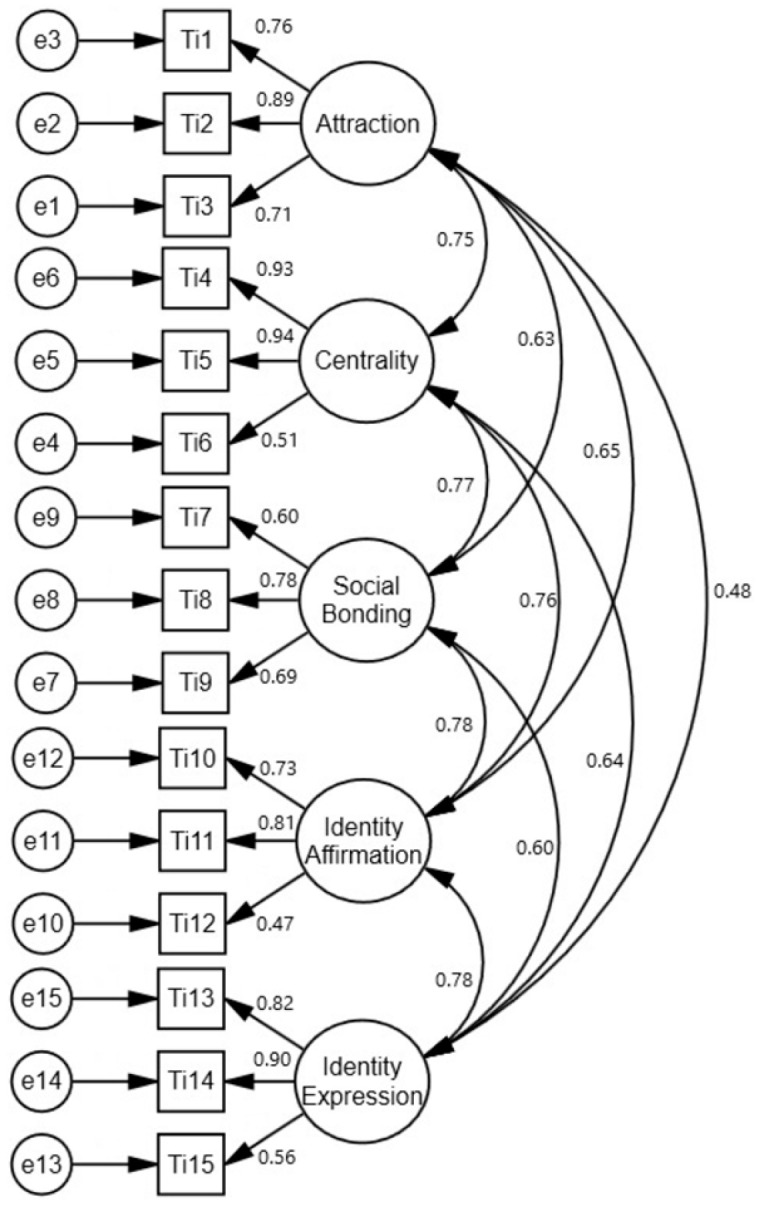
CFA results for the Tennis Involvement Scale.

**Figure 3:**
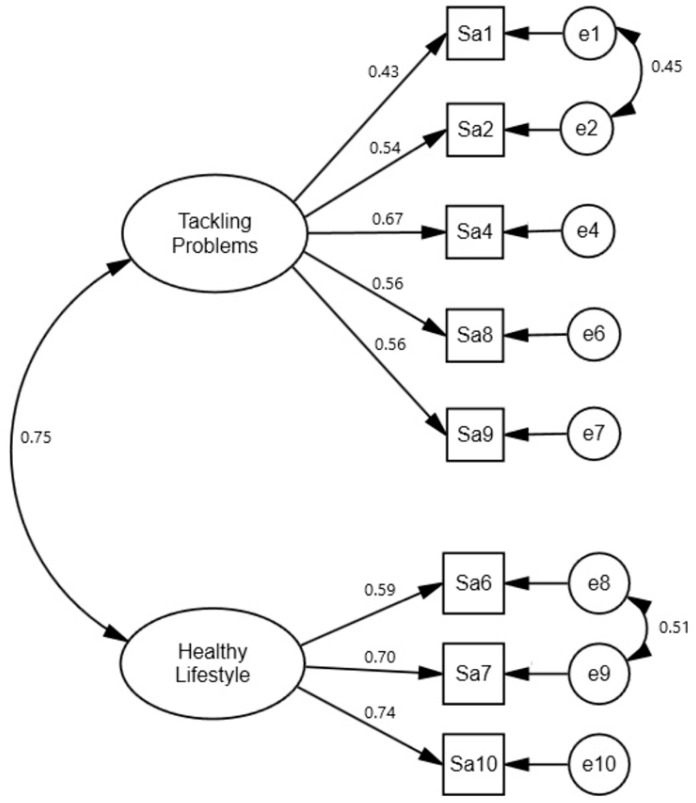
CFA results for the Successful Aging Scale.

Before testing the model created for the study, a CFA was conducted to test the compatibility of the TIS and SAS with the original scales (LIS and SAS) (Figure 2 and Figure 3).

The Tennis Involvement Scale (TIS) shared the same structure as the original scale, with CFA results proving to be statistically significant. The observed variables of the TIS validated the latent variables, with factor loadings ranging from 0.47–0.94. Regression weights were significantly different from zero at the 0.001 level. The TIS comprises five subfactors: “attractiveness,” “centrality,” “social bonding,” “identity affirmation,” and “identity expression.” Each subfactor included three items (observed variables), amounting to a total of 15 items (Figure 2).

When the structure of the Successful Aging Scale, which comprises two subfactors and 10 items, was evaluated using the CFA model, the factor loading for item three in the “Tackling Problems” subfactor was 0.35, and for item five, it was 0.36, both below the threshold of 0.40. Additionally, the values of goodness-of-fit also did not yield the expected results (Table 4). These two items (i3 and i5) were gradually excluded from the analysis because their factor loadings fell below the threshold values (>0.40), and they did not exhibit a good fit. Upon removing these items from the model, a significant improvement in the model fit indices was observed. The newly formed SAS was validated using k-fold cross-validation. CFA results in the exploratory sample (n = 190) showed an acceptable/good fit (CFI = 0.97, RMSEA = 0.05). When this model was independently tested in the validation sample (n = 34), the fit indices were acceptable (CFI = 0.96, RMSEA = 0.08). This indicates that the 8-item structure was stable (Table 4). The new successful aging model for masters tennis players consisted of two subscales (tackling problems, healthy lifestyle) and eight items (Figure 3).

According to the analyses performed for both scales, the new results were found to be within the acceptable range of values [32,33,34]. The standardized path coefficients of all The TIS and SAS items were statistically significant (*p* < 0.001), positive, and above 0.40 (Table 5). When the CR and AVE values were examined, it was observed that the convergent validity of the scales for the model was generally close to ideal values, although some factors were below acceptable limits [35]. However, because the model was at an exploratory level, analyses continued.

### 3.3. Tennis Involvement and Successful Aging Model Analyses

**Figure 4:**
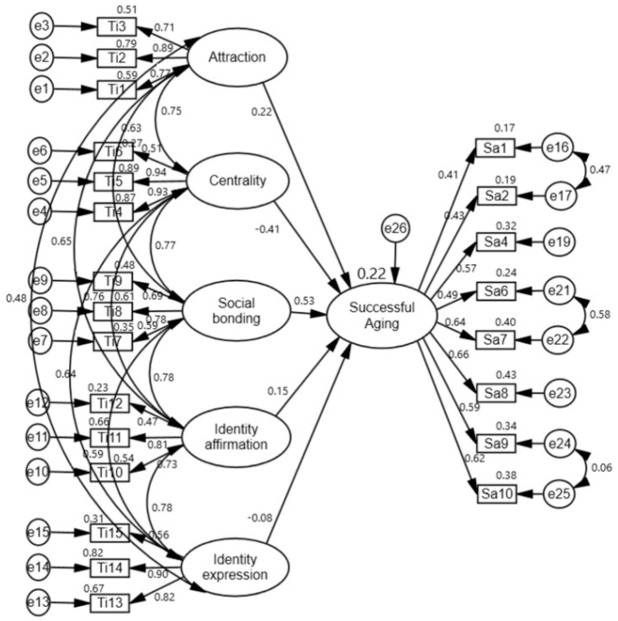
SEM between TI subscales and SA.

**Figure 5:**
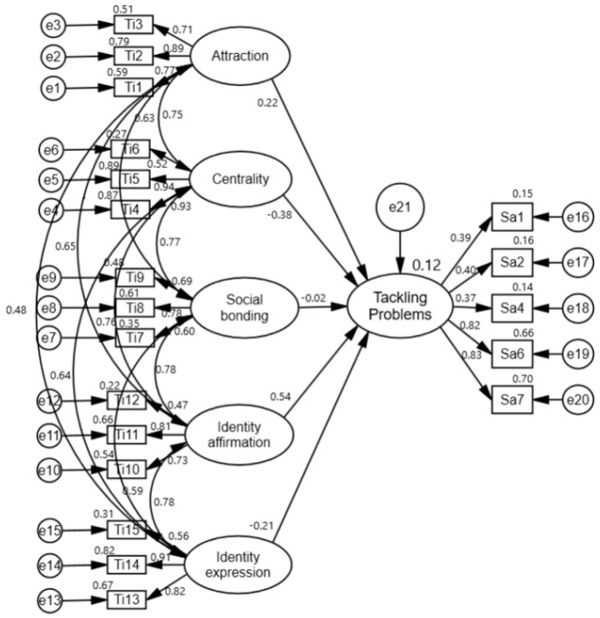
SEM between TI subscales and TP.

**Figure 6:**
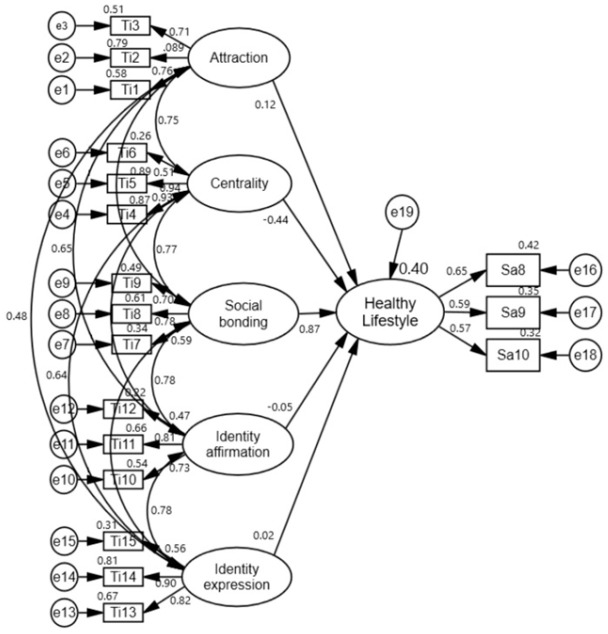
SEM between TI subscales and HL.

To test the research model, SEM analysis was conducted to determine the relationships between tennis involvement subfactors (attraction, centrality, social bonding, identity affirmation, and identity expression) and successful aging subfactors (tackling problems and healthy lifestyle). The results of these analyses are shown in Figure 4, Figure 5 and Figure 6.

In the analyses performed, it was determined that the goodness-of-fit values of the model were χ2(212) = 455.352 (*p* < 0.001), χ2/df = 2.148, CFI = 0.90, IFI = 0.90, and RMSEA = 0.07 for tennis involvement and successful aging; χ2(155) = 382.572 (*p* < 0.001), χ2/df = 2.468, CFI = 0.90, IFI = 0.90, and RMSEA = 0.08 for tennis involvement and tackling problems; and χ2(120) = 272.448 (*p* < 0.001), χ2/df = 2.270, CFI = 0.92, IFI = 0.92, and RMSEA = 0.08 for tennis involvement and healthy lifestyle. Goodness-of-fit values were found to be within acceptable values for tennis interest and successful aging [32,33].

The results of the analysis testing the effects of attraction, centrality, social bonding, identity affirmation, and identity expression, which constitute tennis involvement on successful aging, tackling problems, and a healthy lifestyle, are shown in Table 6.

**Figure 7:**
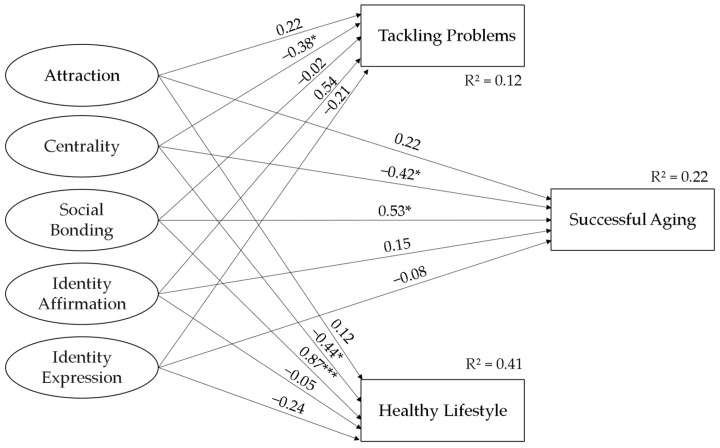
Results related to the model. * *p* < 0.05, *** *p* < 0.001.

Hypotheses H1a, H1b, and H1c predicted that all factors of tennis participation would be positively correlated with Successful Aging, Tackling Problems, and Healthy Lifestyle. SEM analyses provided very limited and partial support for these hypotheses (Figure 7). Only the social bonding factor showed a significant and positive correlation. Hypothesis H1b was completely rejected. Remarkably, however, the centrality factor showed a negative trend, contrary to expectations, and statistically significant correlations were found. The “attraction, centrality, social bonding, identity affirmation, and identity expression” factors of tennis involvement collectively predicted 22% of the variance in “successful aging”, 12% of the variance in “tackling problems”, and 41% of the variance in “healthy lifestyle” (Table 6).

The Pearson correlation analysis was performed to assess the relationships between age, the total days and hours spent playing tennis, and the scale subfactors. The results are presented in Table 7.

The analysis results indicated statistically significant correlations between the age of masters tennis players, the total number of days and hours they played tennis each week, and certain scale factors. Notably, a significant correlation was found only between age and TTD. Additionally, TTD and TTH exhibited a significant positive correlation with each other. In addition to this result, another notable result was the statistically significant positive correlations between TTD and TTH and successful aging, tackling problems, and a healthy lifestyle. Based on this, these results suggest that as middle-aged masters tennis players increased their weekly tennis days and hours, they adopted positive successful aging strategies (Table 7).

## 4. Discussion

This study developed and tested theoretically grounded models to examine associations between tennis involvement and orientations toward successful aging among masters tennis players. The sample, with an average age of approximately 51 years, represents middle-aged regular tennis players who differ from older adult populations typically studied in aging research. Successful aging is conceptualized here as a proactive developmental process, in which individuals adopt behavioral, psychological, and social strategies that may support well-being in later life. It is important to note that the Successful Aging Scale (SAS) in this study assesses predispositions and orientations toward successful aging, rather than measuring actual aging outcomes.

Although prior research has consistently shown that physical activity benefits health and supports successful aging [16,36], little is known about how variations in tennis involvement—such as frequency of play, competition participation, age, and gender—relate to successful aging strategies among masters players. By addressing this gap, the present findings advance understanding of tennis involvement as a meaningful construct and provide insight into its relevance for middle-aged athletes within a developmental perspective on successful aging.

A model was developed to examine the relationships between the subfactors of tennis participation and the components of successful aging. Because the measurement tools were applied to masters tennis players for the first time, CFA was performed on the scales for this research group. Although the structure of the Tennis Involvement Scale was found to be the same as the original scale (LIS), the original structure was not formed for the Successful Aging Scale. As a result of the analyses, the structure of the Successful Aging Scale changed. Therefore, the 8-item scale presented in this study should be considered not as a universal revision of the original Successful Aging Scale, but as a culturally and psychometrically adapted and modified version for use in a specific population (masters tennis players in Türkiye). The removal of items is related to statistical necessities as well as the fact that the “tackling problems” construct is represented in a narrower and more action-oriented way (e.g., active struggle) in this sample. Conceptually, “tackling problems” is intended to assess an individual’s active coping strategies and psychological resilience when faced with challenges. Item 3 (I maintain warm and trusting relations with significant others), which was removed from the scale, identifies a coping resource. However, unlike the other items, it does not directly refer to a coping action or mindset, such as persistence, resilience, or the ability to cope. Similarly, item 5 (I strive to remain independent for as long as possible), which was removed from the scale, while expressing a goal or intention, does not directly reflect the current coping competence, which is the focus of this dimension. Consequently, it aligns less closely with the factor structure. The fact that the removed items (“maintaining close relationships” and “striving to remain independent”) report more of a resource or intention, while the other items emphasize a direct coping action, has served to make this subfactor more consistent while preserving its essence. Thus, the resulting instrument is a measurement tool that continues to measure the core components of successful aging but has been developed with an exploratory approach and optimized for fit to the existing data.

Hypotheses H1a, H1b, and H1c predicted that all five dimensions of tennis participation (attraction, centrality, social bonding, identity affirmation, identity expression) would be positively associated with successful aging and its subfactors. SEM analyses provided very limited and partial support for these hypotheses. The hypotheses were supported only by the Social Bonding factor: Social Bonding showed strong positive associations with Successful Aging and Healthy Lifestyle. In contrast, the centrality factor, contrary to expectations, showed consistently significant negative associations with Successful Aging, Tackling Problems, and Healthy Lifestyle. The relationships between the other factors (attraction, identity affirmation, identity expression) were not found to be significant. These results suggest that the hypotheses require significant revision. In summary, the analysis of the model partially confirmed H1a and H1c, while H1b was rejected.

The positive association between social bonding and successful aging strategies highlights the importance of social support and belonging for individuals around 51 years of age [37], aligning with previous results on the role of social relationships in promoting healthy aging [38,39,40]. Tennis clubs and doubles play may facilitate social network maintenance and engagement during midlife transitions.

Conversely, the negative correlation with centrality should be interpreted cautiously as an observed association rather than a causal effect. Excessive focus on tennis may be related to interference with other life roles and potential increases in competitive stress [41], but this interpretation remains a theoretical hypothesis rather than a demonstrated mechanism. Alternative explanations, such as identity foreclosure or competitive pressure, may also account for these associations. Although high centrality could theoretically align with the Role Overload Theory or U-shaped activity models [41,42,43,44], these models were not directly tested in this study, and no direct measures of stress, burnout, or injury [20,28,45,46,47] were included; therefore, implications regarding training volume or excessive involvement should not be overstated. These results also provide a nuanced view in relation to the Serious Leisure Perspective [48], indicating that strong commitment to tennis may, under certain midlife conditions, transition from supporting identity and mastery to becoming a burdensome role.

Regarding other outcomes, the tennis involvement subfactors “attraction,” “identity affirmation,” and “identity expression” did not show significant associations with successful aging strategies among masters players. Given that participants were over 40 years old with high socioeconomic, educational, and professional status, identity-related experiences may be shaped by broader life factors beyond tennis. Additionally, as active competitors with national and ITF-level goals, these athletes may already engage in behaviors consistent with successful aging orientations, limiting the observable effects of tennis involvement. Future research could further explore these assumptions. Overall, the results suggest that promoting tennis as a social and recreational activity, rather than as a central life focus, may be most supportive of successful aging strategies during midlife.

According to the research results, no correlation was found between age and tennis involvement or successful aging strategies. Although no significant correlation was observed, positive associations emerged between weekly tennis training (both days and hours) and successful aging strategies, indicating that regular participation supports proactive orientations and behavioral strategies relevant to midlife development. These results align with literature emphasizing that regular exercise behavior, particularly in middle-aged populations, is strongly associated with intrinsic motivation (e.g., enjoyment, social connection) and behavioral intention [49]. Furthermore, the adaptable nature of tennis as a recreational sport may account for the minimal impact of chronological age, allowing participants to adjust intensity and style to align with physical capabilities, consistent with Dionigi’s “sport for life” concept [50].

Positive and significant correlations were also observed between weekly tennis training duration, frequency, tennis involvement, and multidimensional successful aging strategies, consistent with evidence that regular exercise benefits physical and psychosocial resources in midlife [15,45]. Tennis involvement may provide a structured and meaningful routine that fosters autonomy, purpose, and competence—central psychological components of successful aging orientations. Its organized nature, including goal setting, social interaction, and skill development, supports persistence and enjoyment, reinforcing the adoption of proactive strategies.

Tennis involvement was positively associated with multiple dimensions of successful aging strategies in middle-aged adults (~51 years), reinforcing the view that successful aging is a proactive, lifelong process [51]. Midlife participation may enhance bone density, physical fitness, cognitive engagement, and social networks, building a “biopsychosocial reserve” that can support adaptive strategies later in life [52,53]. These results underscore the value of promoting tennis as a balanced, socially embedded activity that facilitates proactive orientations toward successful aging.

## 5. Limitations and Strengths

The study’s limitations, particularly in terms of its measurement tools, necessitate caution when interpreting the results. First, the size of the validation subsample is below ideal, which may limit the statistical power of the CFA results. Second, the CR and AVE values obtained for some subscales, which are at or below the recommended thresholds, may indicate that further improvement of the measurement is needed for these constructs. Therefore, claims regarding the construct validity of the scale should be treated cautiously. Finally, the validity and reliability of the scale were tested only with the sample of this study. Future studies are recommended to test this revised form in different and larger samples, to examine test–retest reliability, and to ensure criterion validity (e.g., its relationship with variables such as life satisfaction and depression).

Another limitation of the study is the occurrence of sampling bias. A random and homogeneous sample was used. The sample consisted almost entirely of highly educated individuals, predominantly male, who regularly played tennis and participated in tournaments. These demographic characteristics differ significantly from the profile of the general middle-aged and older population in Türkiye. This creates a selection bias and limits the generalizability of the results to groups with lower levels of education, individuals who do not play tennis, or females. The obtained relationships may also be partly influenced by the resources available to this high-functioning group (time, finances, health awareness).

This study makes several important contributions and presents methodological challenges to existing literature. First, it is one of the pioneering studies examining the concept of successful aging as a proactive process in middle-aged adults (average ~51 years) within the context of physical activity (tennis). This offers a new perspective that treats aging not as a period of ‘loss’ but as a process that can be shaped by active participation.

Second, data were collected from a challenging and unique sample (active masters tennis tournament players), and psychometric properties specific to this group were rigorously assessed through retesting and adaptation of the scales (TIS and SAS). A third strength is the simultaneous testing of complex and direct relationships between the multidimensional nature of tennis involvement and the subcomponents of successful aging, using SEM to examine these relationships.

The most notable result is that the social bonding factor of tennis has the most consistent and strong positive association with successful aging. This is a critical insight into how the benefits of physical activity in middle and old age may stem primarily from psychosocial mechanisms (belonging, social support), rather than purely physiological ones. Furthermore, the unexpected negative correlations of the centrality factor offer a significant practical implication: ‘balance’ and ‘flexibility’ in activity participation may be more harmonious than ‘rigid commitment’. Ultimately, this study repositions tennis not merely as an exercise, but as a meaningful context supporting biopsychosocial health in later life stages.

## 6. Conclusions

In conclusion, among middle-aged players, social and identity-enhancing aspects of tennis involvement are associated with strategies related to successful aging, whereas greater centrality of tennis participation is linked with less favorable psychological outcomes. Higher frequency and longer duration of engagement are also correlated with a healthier lifestyle and problem-solving orientations. These results suggest that, within the context of a flexible and enjoyable tennis practice, participation may be linked to behavioral and psychosocial strategies relevant to successful aging, while emphasizing that these are associations observed in a cross-sectional study rather than causal effects.

## Figures and Tables

**Table 1:** Information and tennis-related characteristics of masters tennis players.

Variable	N	%	M ± SD	*p*
Age	224	100	51.2 ± 8.1	0.000
Female	72	32	48.3 ± 6.5	
Male	152	68	52.6 ± 8.4	
Total Tennis Day	224	100	3.5 ± 1.5	0.004
Total Tennis Hours	224	100	7.4 ± 6.0	0.023
High school	19	9		
University	124	55		
Postgraduate	81	36		

**Table 2:** ITF Age Categories and Tennis-Related Information for Masters Tennis Players.

Age Categories	N	%	TTD M ± SD	TTH M ± SD
40+	58	26	3.2 ± 1.6	7.3 ± 7.3
45+	51	23	3.3 ± 1.6	7.4 ± 5.5
50+	39	17	3.6 ± 1.4	7.7 ± 7.7
55+	33	15	3.6 ± 1.3	6.8 ± 2.9
60+	27	12	4.2 ± 1.7	8.3 ± 5.6
65+	16	7	3.7 ± 1.5	7.2 ± 4.4
			*p* = 0.176	*p* = 0.958

Abbreviations: TTD, total tennis days; TTH, total tennis hours, *p* > 0.05.

**Table 3:** Descriptive statistics and reliability analysis values (n = 224).

Scales/Subscales	Item	M ± SD	Skewness	Kurtosis	Cronbach α
Tennis Involvement ^1–5^	15	3.9 ± 0.7	−0.385	0.030	0.91
Attraction ^1–5^	3	4.6 ± 0.6	−1.452	0.436	0.82
Centrality ^1–5^	3	3.7 ± 1.0	−0.423	−0.663	0.82
Social bonding ^1–5^	3	3.9 ± 0.8	−0.506	−0.161	0.72
Identity affirmation ^1–5^	3	3.7 ± 0.8	−0.255	−0.488	0.71
Identity expression ^1–5^	3	3.6 ± 0.9	−0.435	−0.144	0.79
Successful Aging ^1–7^	8	5.9 ± 0.7	−0.456	−0.389	0.80
Tackling problems ^1–7^	5	5.9 ± 0.7	−0.456	−0.366	0.71
Healthy lifestyle ^1–7^	3	6.0 ± 0.9	−1.040	1.179	0.78

Notes: Item score values 1–5 and 1–7.

**Table 4:** CFA goodness-of-fit and k-fold cross-validation values.

	n	nI	df	χ2	χ2/SD	CFI	IFI	TLI	RMSEA
Tennis Involvement Scale	224	15	80	204.489	2.556	0.93	0.93	0.91	0.08
Successful Aging Scale	224	10	34	112.983	3.320	0.86	0.87	0.82	0.10
Successful Aging Scale ^New^	224	8	23	41.808	1.818	0.96	0.96	0.96	0.06
* Successful Aging Scale ^New^	190	8	23	34.230	1.488	0.97	0.97	0.97	0.05
** Successful Aging Scale ^New^	34	8	23	27.659	1.203	0.96	0.96	0.95	0.08

Abbreviations: nI, number of items; * exploratory sample; ** validation sample; *p* < 0.001.

**Table 5:** Item factor loadings, error terms, and convergent validity values of the scales.

Latent Variables	N	λ	λ^2^	1−λ^2^	CR	AVE
Tennis Involvement	15				0.92 ≥ 0.80	0.57 ≥ 0.50
Attraction	1	0.72	0.51	0.49	0.83 ≥ 0.80	0.63 ≥ 0.60
Attraction	2	0.89	0.79	0.21		
Attraction	3	0.76	0.58	0.42		
Centrality	1	0.51	0.26	0.74	0.85 ≥ 0.80	0.67 ≥ 0.60
Centrality	2	0.94	0.89	0.11		
Centrality	3	0.93	0.87	0.13		
Social bonding	1	0.69	0.48	0.52	0.73 ≥ 0.70	0.48 ≤ 0.50
Social bonding	2	0.78	0.61	0.39		
Social bonding	3	0.60	0.35	0.65		
Identity affirmation	1	0.47	0.22	0.78	0.72 ≥ 0.70	0.47 ≤ 0.50
Identity affirmation	2	0.81	0.66	0.34		
Identity affirmation	3	0.73	0.54	0.46		
Identity expression	1	0.56	0.31	0.69	0.81 ≥ 0.80	0.60 ≥ 0.60
Identity expression	2	0.90	0.81	0.19		
Identity expression	3	0.82	0.67	0.33		
Successful Aging	8				0.82 ≥ 0.80	0.37 ≤ 0.50
Tackling problems	1	0.43	0.18	0.82	0.69 ≤ 0.70	0.31 ≤ 0.50
Tackling problems	2	0.54	0.29	0.71		
Tackling problems	3	0.67	0.45	0.55		
Tackling problems	4	0.56	0.31	0.69		
Tackling problems	5	0.56	0.32	0.68		
Healthy lifestyle	1	0.59	0.34	0.66	0.72 ≥ 0.70	0.46 ≤ 0.50
Healthy lifestyle	2	0.70	0.49	0.51		
Healthy lifestyle	3	0.74	0.55	0.45		

**Table 6:** Hypothesis results for regression coefficients by SA, TP and HL.

H	Relationships Between Parameters		S.E.	C.R.	*β*	*p*	Result (D, M)
H1a	Attraction	→ Successful Aging	0.21	1.47	0.22	0.137	Positive, ns
	Centrality	→ Successful Aging	0.10	−2.13	−0.42	0.033 *	Negative *, ns
	Social Bonding	→ Successful Aging	0.28	2.31	0.53	0.021 *	Positive *
	Identity Affirmation	→ Successful Aging	0.23	0.59	0.15	0.557	Positive, ns
	Identity Expression	→ Successful Aging	0.11	−0.51	−0.08	0.611	Negative, ns
SMC	Tennis Involvement	→ Successful Aging			0.22		
All models explained 22% of the variance in Successful Aging							
H1b	Attraction	→ Tackling Problems	0.20	1.53	0.22	0.127	Positive, ns
	Centrality	→ Tackling Problems	0.10	−1.98	−0.38	0.048 *	Negative *, ns
	Social Bonding	→ Tackling Problems	0.24	−0.10	−0.02	0.921	Negative, ns
	Identity Affirmation	→ Tackling Problems	0.24	1.87	0.54	0.062	Positive, ns
	Identity Expression	→ Tackling Problems	0.11	−1.26	−0.21	0.208	Negative, ns
SMC	Tennis Involvement	→ Tackling Problems			0.12		
All model explained 12% of the variance in Tackling Problems							
H1c	Attraction	→ Healthy Lifestyle	0.28	0.79	0.12	0.430	Positive, ns
	Centrality	→ Healthy Lifestyle	0.14	−2.13	−0.44	0.033 *	Negative *, ns
	Social Bonding	→ Healthy Lifestyle	0.41	3.37	0.87	<0.001	Positive ***
	Identity Affirmation	→ Healthy Lifestyle	0.32	−0.18	−0.05	0.856	Negative, ns
	Identity Expression	→ Healthy Lifestyle	0.15	0.14	0.24	0.893	Positive, ns
SMC	Tennis Involvement	→ Healthy Lifestyle			0.41		
All model explained 41% of the variance in Healthy Lifestyle							

Abbreviations: S.E., standard error; C.R, critical ratio; *β*, standardized regression coefficient; ns, not significant; D, direction; M, meaning; * *p* < 0.05, *** *p* < 0.001.

**Table 7:** Correlations between age, TTD, TTH, TI, SA, and subscales (n = 224).

		TTD	TTH	TI	A	C	SB	IA	IE	SA	TP	HL
Age	r	0.15	−0.01	−0.12	−0.02	0.07	−0.11	−0.12	−0.12	0.05	0.04	0.05
	*p*	0.02 *	0.89	0.08	0.79	0.28	0.12	0.08	0.06	0.47	0.54	0.50
TTD	r	1	0.78	−0.08	0.01	−0.07	−0.09	−0.06	−0.09	0.20	0.17	0.19
	*p*		0.00 **	0.22	0.81	0.30	0.18	0.34	0.17	0.03 **	0.02 *	0.04 *
TTH	r		1	−0.02	−0.00	−0.03	−0.02	−0.00	−0.02	0.19	0.18	0.15
	*p*			0.77	0.99	0.69	0.78	0.95	0.78	0.00 **	0.01 **	0.03 *

Abbreviations: TTD, total tennis day; TTH, total tennis hours; TI, tennis involvement; A, attraction; C, centrality; SB, social bonding; IA, identity affirmation; IE, identity expression; SA, successful aging; TP, tackling problems; HL, healthy lifestyle Significance levels: * *p* < 0.05, ** *p* < 0.01.

## Data Availability

The data that support the findings of this study are available from the first author, upon reasonable request.

## Abbreviations

The following abbreviations are used in this manuscript:

TI	Tennis Involvement
SA	Successful Aging
CFA	Confirmatory Factor Analysis
SEM	Structural Equation Modeling

## References

[1] American College of Sports Medicine (1998). Exercise and physical activity for older adults position stand. Med. Sci. Sports Exerc..

[2] World Health Organization (WHO) (2018). Global Action Plan on Physical Activity 2018–2030: More Active People for a Healthier World.

[3] Republic of Türkiye, Ministry of Health (2022). Türkiye Healthy Living Guide—Physical Activity.

[4] Beard J.R., Officer A., de Carvalho I.A., Sadana R., Pot A.M., Michel J.P., Lloyd-Sherlock P., Epping-Jordan J.E., Peeters G.M.E.E.G., Mahanani W.R. (2016). The World Report on Ageing and Health: A Policy Framework for Healthy Ageing. Lancet.

[5] Gianfredi V., Nucci D., Pennisi F., Maggi S., Veronese N., Soysal P. (2025). Aging, Longevity, and Healthy Aging: The Public Health Approach. Aging Clin. Exp. Res..

[6] World Health Organization (2024). World Report on Ageing and Health.

[7] Young Y., Frick K.D., Phelan E.A. (2009). Can successful aging and chronic illness coexist in the same individual? A multidimensional concept of successful aging. J. Am. Med. Dir. Assoc..

[8] Depp C.A., Jeste D.V. (2006). Definitions and predictors of successful aging: A comprehensive review of larger quantitative studies. Am. J. Ger. Psychiatry.

[9] Rowe J.W., Kahn R.L. (1997). Successful aging. Gerontologist.

[10] Rowe J.W., Kahn R.L. (2015). Successful aging 2.0: Conceptual expansions for the 21st Century. J. Gerontol. B Psychol. Sci. Soc. Sci..

[11] Yazici S. (2018). Perception of successful aging: Views of individuals from different age groups on aging. J. Contin. Med. Educ..

[12] Yi X., Dong X., Gu C., Yi N., Xu B., Liu Q., Gao Z., Gao Y., Zeng H. (2024). Effects of social health and physical activity on emotionality and depression in the elderly: A multiple mediation model: 337. Med. Sci. Sports Exerc..

[13] Shephard R.J., Balady G.J. (1999). Exercise as cardiovascular therapy. Circulation.

[14] Sui X., LaMonte M.J., Laditka J.N., Hardin J.W., Chase N., Hooker S.P., Blair S.N. (2007). Cardiorespiratory fitness and adiposity as mortality predictors in older adults. JAMA.

[15] Chao H.H., Liao Y.H., Chou C.C. (2021). Influences of recreational tennis-playing exercise time on cardiometabolic health parameters in healthy elderly: The ExAMIN Age Study. Int. J. Environ. Res. Public Health.

[16] Lefferts E.C., Ranadive S.M. (2024). Vascular responses to acute induced inflammation with aging: Does fitness matter?. Exerc. Sport Sci. Rev..

[17] Spring K.E., Holmes M.E., Smith J.W. (2020). Long-term tennis participation and health outcomes: An investigation of “lifetime” activities. Int. J. Exerc. Sci..

[18] International Tennis Federation (2024). ITF World Tennis Masters Tour. https://www.itftennis.com/en/itf-tours/itf-world-tennis-masters-tour.

[19] Genevois C. (2019). The importance of aerobic fitness for tennis: A review (Part 1). ITF Coach. Sport Sci. Rev..

[20] Pluim B.M., Groppel J.L., Miley D., Crespo M., Turner M.S. (2018). Health benefits of tennis. Br. J. Sports Med..

[21] Eckstrom E., Neukam S., Kalin L., Wright J. (2020). Physical activity and healthy aging. Clin. Geriatr. Med..

[22] Pan W., Gong L., Xiao G., Zhang L., Xiao Y., Xu C. (2022). Regular tennis exercise may improve the vascular endothelial function in postmenopausal women: The influence of hemodynamics. Int. J. Environ. Res. Public Health.

[23] Schnohr P., O’Keefe J.H., Holtermann A., Lavie C.J., Lange P., Jensen G.B., Marott J.L. (2018). Various leisure-time physical activities associated with widely divergent life expectancies: The Copenhagen city heart study. Mayo Clin. Proc..

[24] Iwasaki Y., Havitz M.E. (2004). Examining Relationships between Leisure Involvement, Psychological Commitment and Loyalty to a Recreation Agency. J. Leis. Res..

[25] Kyle G., Absher J., Norman W., Hammitt W., Jodice L. (2007). A modified involvement scale. Leis. Stud..

[26] Kyle G., Chick G. (2002). The social nature of leisure involvement. J. Leis. Res..

[27] Reker G.T. (2009). A Brief Manual of the Successful Aging Scale (SAS).

[28] Fernandez-Fernandez J., Sanz-Rivas D., Sanchez-Muñoz C., Pluim B.M., Tiemessen I., Mendez-Villanueva A. (2009). Comparison of the activity profile and physiological demands between advanced and recreational veteran tennis players. J. Strength Cond. Res..

[29] Stubbs B., Werneck A. (2024). The Relationship between Tennis Participation and Wellbeing: A Survey of 2287 Adults. Int. J. Racket Sports Sci..

[30] Gurbuz B., Cimen Z., Aydin I. (2018). Leisure involvement scale: Validity and reliability study of Turkish form. Spormetre J. Phys. Educ. Sport Sci..

[31] Hazer O., Özsungur F. (2017). Turkish version of successful aging scale. Int. J. Educ. Technol. Sci. Res..

[32] Kline R.B., Little T.D. (2011). Principles and Practice of Structural Equation Modeling.

[33] Hooper D., Coughlan J., Mullen M.R. (2008). Structural equation modeling: Guidelines for determining model fit. Electron. J. Bus. Res. Methods.

[34] Hu L., Bentler P.M. (1999). Cutoff criteria for fit indexes in covariance structure analysis: Conventional criteria versus new alternatives. Struct. Equ. Model..

[35] Fornell C., Larcker D.F. (1981). Evaluating structural equation models with unobservable variables and measurement error. J. Mark. Res..

[36] Warburton D.E., Nicol C.W., Bredin S.S. (2006). Health benefits of physical activity: The evidence. Can. Med. Assoc. J..

[37] Umberson D., Karas Montez J. (2010). Social relationships and health: A flashpoint for health policy. J. Health Soc. Behav..

[38] Marks B.L. (2006). Health benefits for veteran (senior) tennis players. Br. J. Sports Med..

[39] An H.Y., Chen W., Wang C.W., Yang H.F., Huang W.T., Fan S.Y. (2020). The relationships between physical activity and life satisfaction and happiness among young, middle-aged, and older adults. Int. J. Environ. Res. Public Health.

[40] Lara R., Vázquez M.L., Ogallar A., Godoy-Izquierdo D. (2020). Psychosocial resources for hedonic balance, life satisfaction and happiness in the elderly: A path analysis. Int. J. Environ. Res. Public Health.

[41] Goode W.J. (1960). A Theory of Role Strain. Am. Sociol. Rev..

[42] Huang Q., Wang Y., Yuan K., Liu H. (2022). How Role Overload Affects Physical and Psychological Health of Low-Ranking Government Employees at Different Ages: The Mediating Role of Burnout. Saf. Health Work.

[43] Zhou H., Jiang F., Liu H., Wu Y., Tang Y.L. (2025). Dose-Dependent Association between Physical Activity and Mental Health, and Mitigation Effects on Risk Behaviors. iScience.

[44] Gallardo-Gómez D., del Pozo-Cruz J., Pedder H., Martínez-Torres J., del Pozo-Cruz B., Espinoza C., Kuo T., Peña F., Luo M., del Pozo-Cruz J. (2023). Optimal Dose and Type of Physical Activity to Improve Functional Capacity and Minimise Adverse Events in Acutely Hospitalised Older Adults: A Systematic Review with Dose–Response Network Meta-Analysis of Randomised Controlled Trials. Br. J. Sports Med..

[45] Gopinath B., Kifley A., Flood V.M., Mitchell P. (2018). Physical activity as a determinant of successful aging over ten years. Sci. Rep..

[46] Feng Z., Cramm J.M., Nieboer A.P. (2019). A healthy diet and physical activity are important to promote healthy ageing among older Chinese people. J. Int. Med. Res..

[47] Achour E.C., Barthelemy J.C., Lionard K.C., Trombert B., Lacour J.R., Thomas-Anterion C., Gonthier R., Garet M., Roche F. (2011). Level of physical activity at the age of 65 predicts successful aging seven years later: The PROOF study. Rejuvenation Res..

[48] Stebbins R.A. (2020). The Serious Leisure Perspective: A Synthesis.

[49] Winzer E.B., Woitek F., Linke A. (2018). Physical activity in the prevention and treatment of coronary artery disease. J. Am. Heart Assoc..

[50] Dionigi R. (2006). Competitive sport as leisure in later life: Negotiations, discourse, and aging. Leis. Sci..

[51] Baltes P.B., Baltes M.M. (1990). Psychological Perspectives on Successful Aging: The Model of Selective Optimization with Compensation; Successful aging: Perspectives from the behavioral sciences.

[52] Ducher G., Courteix D., Mémoire S., Magni C., Viala J.F., Benhamou C.L. (2005). Bone geometry in response to long-term tennis playing and its relationship with muscle volume: A quantitative magnetic resonance imaging study in tennis players. Bone.

[53] Verghese J., Lipton R.B., Katz M.J., Hall C.B., Derby C.A., Kuslansky G., Ambrose A.F., Sliwinski M., Buschke H. (2003). Leisure activities and the risk of dementia in the elderly. N. Engl. J. Med..

